# A novel methodology to study antimicrobial properties of high-touch surfaces used for indoor hygiene applications—A study on Cu metal

**DOI:** 10.1371/journal.pone.0247081

**Published:** 2021-02-25

**Authors:** T. Chang, M. Sepati, G. Herting, C. Leygraf, G. Kuttuva Rajarao, K. Butina, A. Richter-Dahlfors, E. Blomberg, I. Odnevall Wallinder

**Affiliations:** 1 Department of Chemistry, KTH Royal Institute of Technology, Div. Surface and Corrosion Science, School of Engineering Sciences in Chemistry, Biotechnology and Health, Stockholm, Sweden; 2 AIMES—Center for the Advancement of Integrated Medical and Engineering Sciences at Karolinska Institutet and KTH Royal Institute of Technology, Stockholm, Sweden; 3 Department of Neuroscience, Karolinska Institutet, Stockholm, Sweden; 4 Department of Chemistry, Materials and Chemical Engineering "Giulio Natta", Politecnico di Milano, Milan, Italy; 5 Department of Industrial Biotechnology, School of Engineering Sciences in Chemistry, Biotechnology and Health, KTH Royal Institute of Technology, Stockholm, Sweden; 6 KTH Royal Institute of Technology, School of Engineering Sciences in Chemistry, Biotechnology and Health, Fibre and Polymer Technology, Stockholm, Sweden; University of Vigo, SPAIN

## Abstract

Metal-based high-touch surfaces used for indoor applications such as doorknobs, light switches, handles and desks need to remain their antimicrobial properties even when tarnished or degraded. A novel laboratory methodology of relevance for indoor atmospheric conditions and fingerprint contact has therefore been elaborated for combined studies of both tarnishing/corrosion and antimicrobial properties of such high-touch surfaces. Cu metal was used as a benchmark material. The protocol includes pre-tarnishing/corrosion of the high touch surface for different time periods in a climatic chamber at repeated dry/wet conditions and artificial sweat deposition followed by the introduction of bacteria onto the surfaces via artificial sweat droplets. This methodology provides a more realistic and reproducible approach compared with other reported procedures to determine the antimicrobial efficiency of high-touch surfaces. It provides further a possibility to link the antimicrobial characteristics to physical and chemical properties such as surface composition, chemical reactivity, tarnishing/corrosion, surface roughness and surface wettability. The results elucidate that bacteria interactions as well as differences in extent of tarnishing can alter the physical properties (e.g. surface wettability, surface roughness) as well as the extent of metal release. The results clearly elucidate the importance to consider changes in chemical and physical properties of indoor hygiene surfaces when assessing their antimicrobial properties.

## Introduction

According to the World Health Organization (WHO), antibiotic resistance is today one of the primary threats to global health and food safety, with longer hospital stays, higher medical costs and increased morbidity and mortality as a few examples of consequences [1]. Indoor hygiene (IH) surfaces, especially high-touch surfaces such as door handles and knobs in hospitals, in commercial, residential, and healthcare settings are recognized as possible reservoirs of infectious agents and potential spreader of multi-resistant organisms, increasing the infection risk [2, 3]. Recent findings show further that the use of modern jet-air dryers as hand drying methods in e.g. hospital washrooms generate aerosols that spread bacteria onto different touch surfaces, hence increasing rather than reducing the risk for infection [4]. Similar dryers are today commonly used in different public settings such as rest rooms at train stations, airports and shopping centers. This emphasizes the use of high-touch surfaces that intrinsically possess antimicrobial properties able to hinder/reduce bacteria spreading. In recent years, copper (Cu) metal and Cu-based alloys surfaces are increasingly used to reduce the occurrence of healthcare-associated infections related to high-touch surfaces [5–10]. Other materials include e.g. stainless steel, silver- and ZnO-containing coatings [5, 11, 12].

Due to their desirable intrinsic antimicrobial efficiency, Cu metal and Cu-based alloys have gained increased interests within the antimicrobial community as a potent material for furnishing IH spaces [8, 13–17]. This has been more evident since the U.S. Environmental Protection Agency (EPA) approved their ability to inactivate or kill 99.9% pathogenic bacteria within two hours [18, 19]. Although the antimicrobial mechanisms of Cu are not fully understood, Cu ions released from the Cu surface are largely believed to play a key role for inducing bacterial death [16, 20–22]. The infection chain in bacteria-favored environments has been shown to efficiently break via contact with Cu-based surfaces as a result of a considerable reduction in the number of bacteria, a process known as “contact killing” [22–27]. This remarkable reduction in number of microorganisms has been reported in studies both at laboratory conditions and at real settings in hospitals [28]. Other studies at clinical settings show only modest microbial reductions [6]. Controversial findings of the antimicrobial efficiency of Cu are suggested to mainly relate to inconsistent test conditions of various standards, such as relative humidity and temperature [29, 30], wet or dry applications [19, 31, 32], different bacterial strains [19, 32, 33], and chemical environments [34, 35].

Oxidation/corrosion reactions take place on metallic surfaces at indoor conditions evolving different surface oxides or more complex corrosion products depending on the prevailing environmental- (e.g. relative humidity, temperature) and pollutant conditions (air constituents, e.g. CO_2_, O_3_, SO_2_, NO_2_, NH_3_, HCHO, HCOOH, Cl^−^(e.g. from NaCl or NH_4_Cl)). Depending on the characteristics of evolved corrosion products, the extent of released metal ions into the aqueous adlayer will differ as will the surface roughness, aspects of relevance for the antimicrobial properties of any metallic surface [36]. For high-touch surfaces, finger contact results in the additional transfer of corrosive species (e.g. sweat with chlorides, fatty acids, bacteria) to the metallic surface that is exposed to daily cycles of dry and wet conditions. Such conditions should be considered in laboratory tests that aim to mimic finger-print contact at indoor environments [37].

Cu metal forms Cu(I)oxide (cuprite, Cu_2_O) at indoor ambient atmospheric conditions. At high temperatures and/or in the presence of strong oxidants, cuprite can partially be transformed into Cu(II)oxide (tenorite, CuO) [38]. Literature findings on contact killing claim Cu_2_O to possess improved antimicrobial properties compared with CuO [35]. However, changes in the antimicrobial property of a Cu metal surface with oxides and/or other corrosion products formed in contact with artificial sweat at dry/wet atmospheric indoor aqueous thin film conditions have not previously been investigated even though corrosion studies have been performed at immersion conditions in synthetic perspiration [39–41].

A variety of test methods have been suggested to inspect the antimicrobial efficacy of massive surfaces of Cu metal and Cu-based alloys [18, 32, 42–46] at different relative humidities, temperatures, inoculum solutions, volume to surface area ratios, and microbial cultures *etc* [7]. Existing protocols are based on investigations in a nutrient broth ideal for the growth of bacteria [47] rather than in contact with human or synthetic sweat, as would be a more realistic case. A recent US patent describes immersion tests in artificial perspiration as an approach to define the antimicrobial killing efficacy of a Cu alloy (Cu-Al-Sn) against *Escherichia coli* (*E*. *coli)* [46]. The patent suggests immersion of a 1.1 cm^2^ alloy surface in 200 mL artificial perspiration containing 2×10^6^ colony forming units (CFU) of *E*. *coli*. Compared to complete immersion conditions, this method better mimics the real exposure conditions with hand/finger contact. Nevertheless, the synthetic perspiration with a volume of 200 mL is more likely to simulate very wet conditions that seldom exist on high-touch surfaces at indoor atmospheric conditions. Such settings are better described with apparent dry surfaces, i.e. the presence of only very thin aqueous films or local droplets that will result in a different corrosion behavior of the metallic surface compared to immersion and wet conditions [38].

In order to evaluate the antimicrobial efficacy of metallic surfaces used for IH-applications it is hence essential to mimic a realistic exposure scenario as closely as possible also from a corrosion/oxidation perspective. In this study, a novel laboratory methodology is presented that mimics surface oxidation/corrosion of IH surfaces at indoor conditions via daily changes in relative humidity at constant temperature and repeated deposition of artificial sweat aerosol droplets for exposure periods from one day up to 4 weeks. A protocol on how to assess the microbial viability was elaborated in parallel and employed for differently oxidized (aged) surfaces. The test strategy is illustrated with results obtained for Cu metal and its antimicrobial effects on *Escherichia coli* bacteria. This bacterial strain was selected since it is easy to handle at laboratory conditions (biosafety level 1) and an identified constituent on high-touch surfaces in e.g. the health care sector [48–50].

Generated results on Cu metal can be used as a benchmark when testing other antimicrobial surfaces intended for IH applications. The killing efficiency with time was explored for differently corroded surfaces in relation to Cu release, surface appearance, -roughness and -wettability.

## Experimental

### High-touch surfaces

Commercially available Cu metal (DPH-Cu, purity 99.98%) was kindly provided by Aurubis, Finland. These as-received Cu metal surfaces were ultrasonically cleaned in analytical grade acetone and isopropanol (each solvent for 5 min) and subsequently dried by cold nitrogen gas, before aged at different conditions and time periods in a climate chamber to mimic indoor corrosion/oxidation, see below. Commercial microscope glass slides (VWR) were cut to the requested dimension (1 cm^2^) using a diamond tipped glass cutter and used as reference surfaces.

#### Artificial sweat and pre-deposition on high-touch surfaces

Fingerprint contact was simulated by using artificial sweat (ASW) prepared according to the EN 1811 standard [51]. The ASW solution was made by mixing 5.0 g/L sodium chloride (NaCl), 1.0 g/L urea (CH_4_N_2_O), 1.0 g/L lactic acid (C_3_H_6_O_3_), and ultrapure water (Milli-Q, 18.2 MΩ·cm). The final solution pH was 6.5 ± 0.05 (adjusted by adding NaOH). ASW was freshly prepared and used within 8 h of preparation to pre-deposit in a well-controlled way using an air brush (Aztek-A220 Broad Stroke Airbrush system) on the high-touch surfaces.

#### Climatic chamber exposure to mimic indoor atmospheric oxidation/corrosion

**Fig 1:**
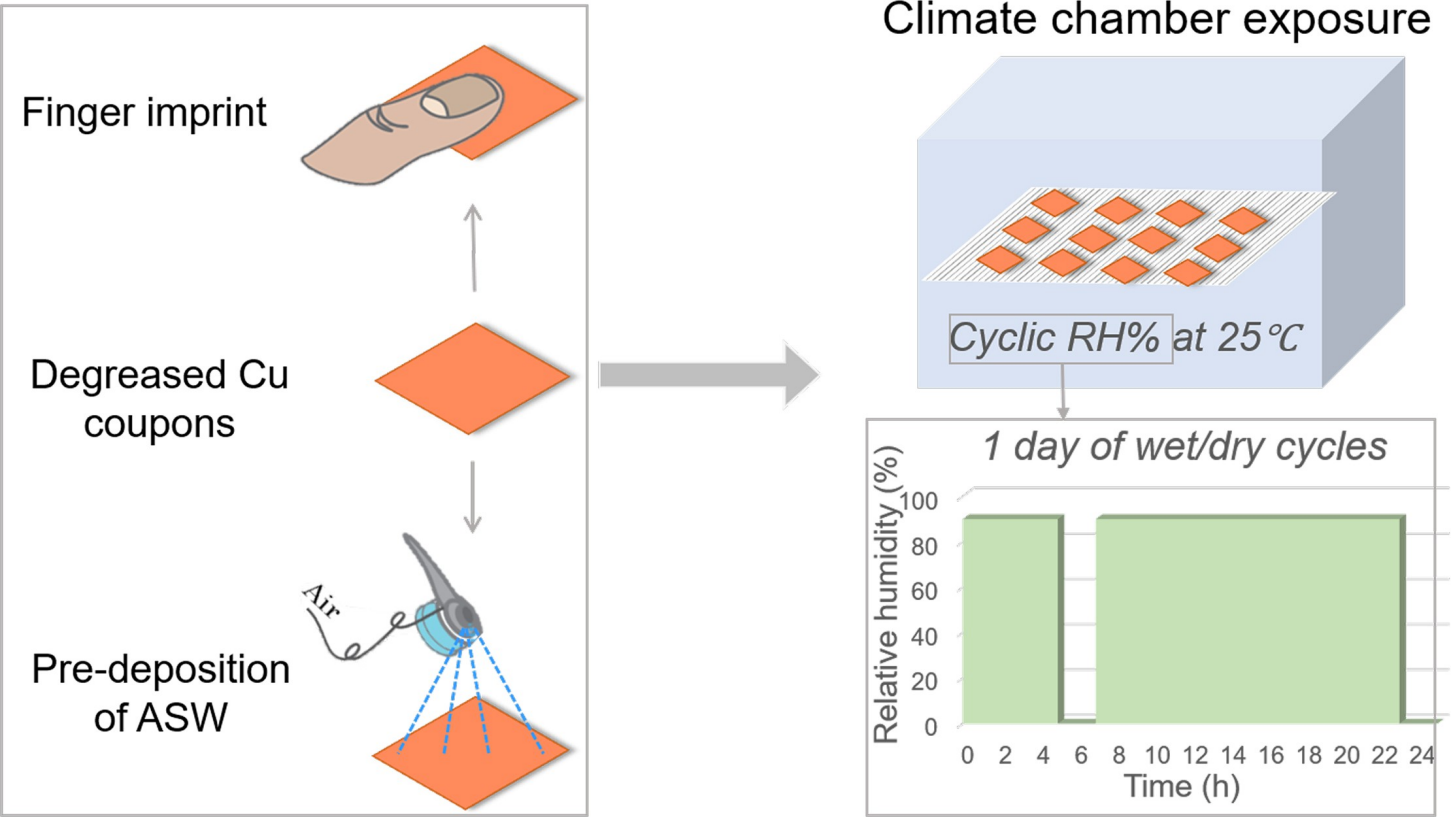
Illustration of sample preparation and the wet/dry cyclic laboratory exposure conditions in a climate chamber.

To mimic and accelerate indoor atmospheric corrosion conditions, Cu surfaces with and without pre-deposited ASW were introduced in a climate chamber and exposed to repeated dry and wet daily cycles (1^st^ cycle: 1–4 h (RH 90%), 2 h (RH 0%); 2^nd^ cycle: 2–16 h (RH 90%), 2 h (RH 0%)) at constant temperature (25°C), see Fig 1. Coupons were withdrawn after 1 day, 1 week, 2 weeks and 4 weeks.

### Bacterial strain, culture medium and viability testing

A gram negative, *Escherichia coli* (*E*. *coli K12*) bacterial strain was used as model organism to provide insight on the antimicrobial properties of IH surfaces and enable laboratory studies at a biosafety level 1. *E*. *coli* was grown overnight in nutrient broth (NB) medium at 37°C. The culture was centrifuged at 2500 rpm for 5 min, the cell pellet was washed with filter sterilized ASW (0.2 μm syringe filter) and repeated the centrifugation to collect the cell pellet. The cells were suspended in ASW followed by dilution to the desired optical density, OD_600_ of (0.1, 0.5 or 1.0) using a UV-Vis spectrophotometer (Varian). ASW was used as the bacterial inoculum to mimic finger-print contact with a high-touch surface.

Nutrient agar (NA)-plates were prepared in 1 L glass bottles using 500 mL deionized water, 7.5 g Agar powder (VWR, BDH chemicals) and 4 g nutrient broth (meat extract (1.5 g) and peptone (2.5 g)) and autoclaved at 121°C for 15 min using steam pressure (15 psi). The sterilized NA medium was poured onto sterile petri plates and allowed to solidify at room temperature followed by storage under refrigerated conditions until further used. The NA-plates were used to quantify the viable number of bacteria and/or colonies per mL detached via vortexing from duplicate surfaces of bacteria-incubated Cu coupons of different extent of tarnishing/oxidation. The number of colony forming units (CFU/mL) was estimated after 24 h.

Trypic Soy Broth (Sigma Aldrich, Sweden) and Tryptic Soy Agar (Sigma Aldrich, Sweden) were prepared according to instructions of the manufacturer. The *E*. *coli* ATCC 25922 strain (Oxoid, United Kingdom) was used in the live-dead assay. Bacteria were maintained as glycerol stocks at -80°C and streaked on Tryptic Soy Agar before the experiment. Bacterial cultures were prepared by inoculating 10 mL Tryptic Soy Broth and incubated for approx. 12.5 h at 37°C under shaking (160 rpm) conditions.

#### Live and dead bacteria staining

Any presence of live bacteria on the surface present was investigated by means of a live/dead staining method (Live-Dead Bacterial Viability Kit L7012, Thermo Fisher Scientific, Waltham, MA USA) assessing cytoplasmic membrane permeability properties damage. The method uses a dye, propidium iodide (PI) that, if the membranes are damaged and permeable, enters the cells and stains cellular DNA (and thus the cells). Bacterial cultures were washed and resuspended in sterile ASW and the OD600 was adjusted to 0.9 ± 0.1. Bacteria were sprayed on glass (autoclaved, reference) and Cu surfaces using spray bottles (purchased from Apotek Hjärtat, Sweden) from approximately a 20 cm distance. After spraying, bacteria were incubated on the surfaces for 0 min and 20 min at ambient conditions. The incubation time was counted as the time before staining, However, since the preparation time was 10–15 min before imaging, the actual incubation time was slightly longer. Following incubation, the surfaces were flipped on a drop (7–10 μL) of Live-Dead stain solution (30 μM Propidium Iodide, 5 μM SYTO9 in 0.9% NaCl) placed on a 24 mm × 50 mm cover glass (VWR, Sweden). The surfaces were sealed on the cover glass using nail polish. Confocal microscopy (Olympus FV1000) was performed at Biomedicum Imaging Core facility (Karolinska Institutet, Sweden) imaged using an Olympus FV1000 instrument with a 40× objective. A 473 nm excitation laser was employed to visualize SYTO9. Emission was collected at 490–540 nm. For Propidium Iodide, emission was collected at 575–675 nm using a 559 nm excitation laser. Four or five images were acquired on each surface at random areas, and each imaging was performed three times on separate samples (representative images presented below). The Image J imaging software was used to process all images [52].

### Elaboration of test approach to assess the antimicrobial property of IH-surfaces (Cu metal surface as a benchmark)

**Fig 2:**
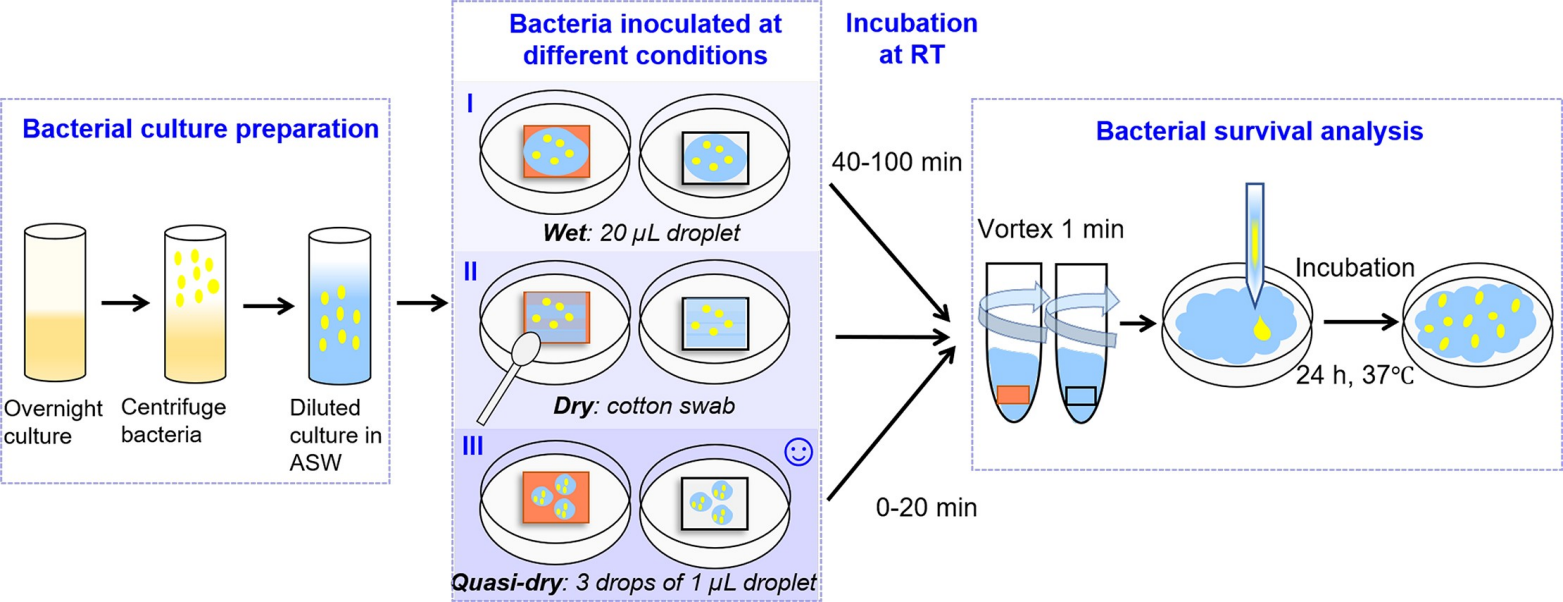
Schematic illustration of the three methodological approaches performed to assess the antimicrobial properties of high touch surfaces of relevance for indoor atmospheric conditions (Cu metal used as a benchmark surface).

New protocols were elaborated in order to efficiently study the antimicrobial properties of IH-surfaces at simulated skin-to-surface contact conditions, schematically illustrated in Fig 2.

*E*. *coli (K12)* was grown overnight in NB medium at 37°C. The bacterial cells were collected after centrifugation, washed with filter-sterilized ASW followed by suspending the cell pellet in ASW adjusting the cell density to OD_600_ = 0.5. ASW was used as the bacterial medium to mimic realistic conditions of high-touch surfaces.

**Fig 3:**
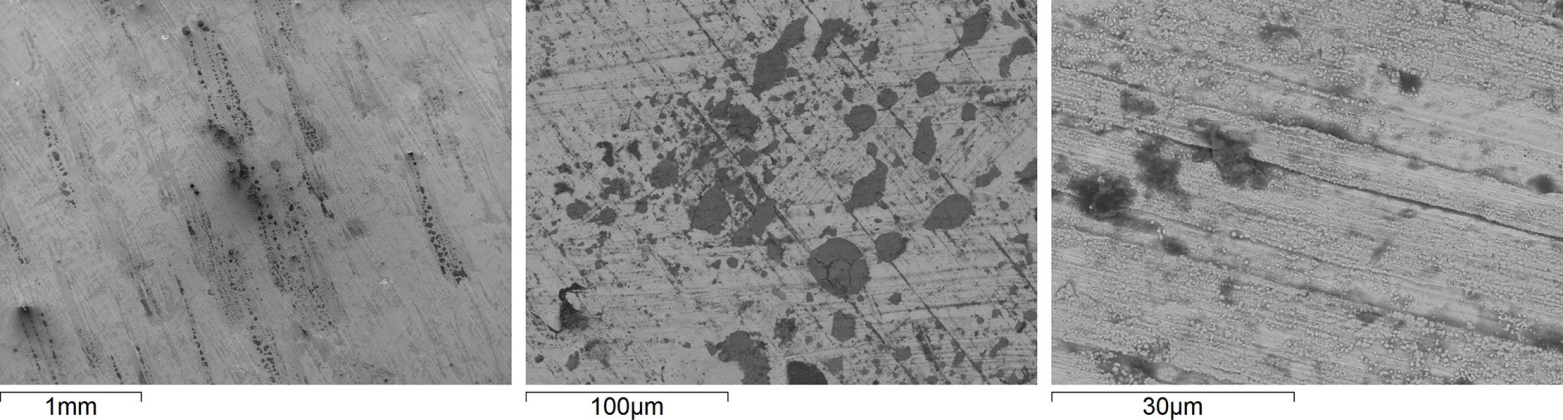
SEM-BE images of locally occurring corrosion features formed on three Cu metal surfaces exposed to daily fingerprint contact (thumb imprint) for one week.

Bacterial culture was deposited onto Cu metal and glass surfaces in different ways to establish a reproducible way to study the antimicrobial properties. Two protocols (Fig 3) (wet and quasi-dry) were established based on extensive experimental testing [53] and information provided in standards [30, 47, 54] and in the scientific literature [24, 28, 33, 55]. The *wet protocol* is based on the procedure described elsewhere [55]. A volume of 20 μL ASW containing *E*. *Coli* (OD_600_ = 0.5) was inoculated in 5 drops with a pipette and spread over the 1 × 1 cm^2^ tarnished/corroded Cu surfaces and control glass surfaces. This was immediately followed by incubation at room temperature (RT) for different time periods (between 0–100 min). The procedure was repeated three times. However, since this procedure rather reflects aqueous bulk conditions than thin film conditions (relevant for high-touch surfaces) and that it takes 65 ± 15 min for complete drying, another methodology was elaborated.

In the second approach, denoted the *dry protocol*, bacteria were transferred to the test surface using a cotton swab. The approach was based on the procedure described elsewhere [56]. The sterile cotton swab was soaked with ASW containing bacteria (*E*. *coli*, OD_600_ = 1,) immersed in 50 μL, 100 μL or until the cotton swab was completely soaked to saturation before transfering the bacteria to the Cu surfaces. The swab was kept in a certain orientation and dragged in a defined zig-zag manner on the sample surface for 3 seconds and subsequently immediately placed in 2 mL ASW.

In the third approach, denoted the *quasi-dry protocol*, 3 tiny droplets (1 μL, covering most of the surface without immediately coalescing) of ASW containing *E*. *coli* (OD_600_ = 1) were manually deposited onto each surface (1 × 1 cm^2^ sized surfaces of Cu metal (three replicates)) using a pipette. This deposition was followed by incubation at RT for 0 to 20 min. As the droplet more readily spreads and forms a thin aqueous layer, this approach was considered more realistic for the indoor conditions and hence more similar to the dry conditions of high touch surfaces in service and as described in the literature [24, 57].

Different ways to detach bacteria from the differently oxidized and bacteria-incubated surfaces were investigated including immersion in 2 mL ASW (no stirring), 1 min vortexing (full speed), 1 min vortexing with glass beads (full speed), bath sonication for 30 sec and the addition of 3 mM SDS (sodium dodecyl sulfate) mixed with 2 mL ASW for 30 min using a rotator. Since all results except SDS (which reduced the number of viable *E*. *coli* bacteria) resulted in similar results, vortexing was selected as the detachment method.

The differently oxidized and bacteria-incubated surfaces were inserted into sterile falcon tubes (polypropylene, PP) containing 2 mL ASW and vigorously vortexed for 1 min at high speed to enable loosely attached bacteria to detach from the surfaces. 100 μL the vortexed solution was after serial dilution deposited onto a Nutrient agar plate and incubated for 24 h at 37°C to determine the presence of live bacteria.

### Surface characterization

#### Surface appearance

Changes in surface appearance upon exposure of the oxidized Cu surfaces with and without pre-deposited ASW were quantitatively assessed by using spectrocolorimetry (Minolta CM-600D spectrophotometer) and D65 light. Presented results reflect average values based on 3 measurements on each coupon. Relative colorimetry changes in appearance are described according to the three-coordinate CIELab color reference space (a* (red/magenta-green), b* (yellow/blue) and L* (lightness—black to white)) and as the total variation in color ($\Delta E=\sqrt{({\Delta L}^{*}{)}^{2}+({\Delta a}^{*}{)}^{2}+({\Delta b}^{*}{)}^{2}}$). More details are given in [58, 59].

#### Surface wettability

Contact angle measurements were conducted to examine changes in surface wettability of the Cu metal surfaces as a function of ageing/corrosion by using a PGx Potable Contact Angle Measurement Meter (FIBRO System AB, Sweden). An ASW droplet (2–5 μL) was illuminated when dispensed on the surface and immediately captured by a high-resolution CCD camera. The contact angle was determined as the average value of 3 measurements.

#### Surface morphology

SEM/EDS (Scanning electron microscopy and energy dispersive spectroscopy) analyses were conducted to obtain surface morphological and elemental information of top-surfaces and cross-sections of the Cu metal coupons corroded with and without pre-deposited ASW and/or seeded bacteria. All images were acquired by means of FEI-XL 30 series instrument equipped with an Oxford X-Max SDD (Silicon Drift Detector) 20 mm^2^ EDS system using secondary electrons (SE) at accelerating voltages of 5 kV and 15 kV for surfaces with and without bacteria, respectively.

#### Surface reactivity—copper release in artificial sweat

Duplicate Cu metal coupons sized 1.5 × 1.5 cm^2^ (with back sides and edges sealed with nail polish) were completely immersed in 3.5 mL fresh ASW at tilted (45°) conditions in 10 mL large exposure vessels (clear glass with PE snap caps). Immersions were conducted at 30°C at dark conditions in an incubator for 4, 24 and 168 h following the EN1811 standard [51].

In order to mimic the touch condition (quasi-dry), the extent of Cu release was analyzed by collecting the solution from the small (3 μL in total) ASW droplets with and without the presence of *E*. *Coli* (OD_600_ = 0.1). The results were compared with immersion condition. To obtain a sufficient volume and Cu concentration for the analysis, 192 droplets (each 3 μL) were carefully pipetted using a micro-pipette onto triplicate surfaces of fresh Cu metal. The droplets were collected after 10 min of contact ensuring that the tip of the pipette did not touch the surface.

Blank reference solutions (ASW only) were exposed in parallel and used to correct for background concentrations of Cu for each condition. Prior to analyses of total amounts of released Cu, the test solutions were digested with acid to a pH<2 (20 μL 65% HNO_3_). Total concentrations of released Cu were analyzed by means of flame Atomic Absorption Spectroscopy (AAS) using a PerkinElmer AA800 analyst instrument operated at standard conditions. Calibration standards were prepared using 0, 1, 3, 10, 30 and 66 mg Cu/L, and quality control samples were run every 6^th^ sample. Limits of detection (LOD) and quantification (LOQ) were 0.020 and 0.050 mg Cu/L respectively. Triplicate readings were made for each sample.

## Results

### Finger imprint–Pilot study

Different indoor surfaces are inevitably touched by people on a daily basis. This may both result in the transfer of corrosive species, e.g. chlorides, lactic acid and fatty acids from sweat to the surface, but also in the possible transfer of different pathogens such as bacteria, both to and from the surface. An initial pilot study was conducted including three persons of different gender, habits and ethnicity to investigate the effect of daily (during the work week) fingerprint contact (same time every day during lab work) with a Cu metal surface exposed to daily wet/dry cycles in a climatic chamber during 4 weeks. The thumb of each individual was, in sequence, daily pressed (each person aiming to apply similar pressure every time) on an agar plate and three Cu metal surfaces in sequence followed by an agar plate with the aim to both assess the effect of fingerprint contact on the corrosion behavior, and to provide a relative measure on the number of bacteria that possibly can be transferred from the thumb to and from a high-touch surface of Cu metal. Fingerprint contact clearly transferred different constituents (mainly composed of C, O, N, Na, Cl, determined by means of EDS) to the surface, which with time resulted in locally distributed corrosion features, Fig 3. The imprint findings further showed that bacteria were clearly transferred from the thumb to the agar plates, in average 70 colony forming units (CFU) per fingerprint, varying between 10 and 170. No viable bacteria were observed after 24 h of incubation either when the finger-print exposed Cu metal coupons were imprinted on NA plates or when detached from the exposed surfaces via vortexing and subsequent incubation.

The results of the pilot study in terms of appearance and formation and evolution of the fingerprint-induced local corrosion products were used as a bench mark in the process to develop a methodology able to mimic finger-print contact with high-touch surfaces at indoor atmospheric corrosion conditions.

### Elaboration of a test methodology to assess indoor corrosion (tarnishing/corrosion) and antimicrobial characteristics of indoor high-touch surfaces

#### Simulated indoor atmospheric corrosion of Cu metal as a benchmark for a high-touch surface

**Fig 4:**
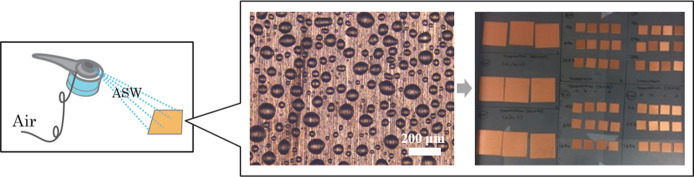
Illustration of deposited ASW droplets (scale bar 200 μm) sprayed by means of an air-brush (left) onto several Cu coupons (middle) simultaneously for further exposure (right) to cyclic wet/dry exposure conditions in a climatic chamber for 1 day to 4 weeks.

Since the fingerprint contact with high touch surfaces transfers and distributes corrosive species unevenly to a surface and with low reproducibility, a general methodology was elaborated to simulate this transfer in a well-defined and reproducible way. Several approaches were explored to evenly distribute tiny droplets of ASW that readily evaporated from the surface. Trials with a sponge and a micropipette applying small droplets (50 μL) on the surface were unsuccessful since the droplets coalesced in an uneven way due to poor wetting properties of the surfaces. In addition to generating non-reproducible results, these methodologies were very time-consuming and hence not applicable for its purpose. Time-efficient and reproducible results were instead accomplished by depositing a fine aerosol of ASW onto the touch surfaces by using an air brush, Fig 4. After optimization of the air pressure, the gap of the nozzle, the distance between the test coupons and the nozzle, as well as the orientation of the coupons vs. the nozzle. The method generated a relatively even and reproducible surface distribution of small sized droplets (10–150 *μm*) with a mean deposited total mass of 6.8 ± 0.8 mg ASW/cm^2^ (based on 6 repeated measurements). The approach enabled simultaneous deposition of ASW on a large number of coupons (sized 1×1 cm^2^) and as droplets that visually rapidly formed a thin aqueous layer.

According to literature findings, the average sweat rate from the palms of humans typically equals 0.1 mg/cm^2^, min (0.2–0.4 mg/cm^2^, min during exercise) [60]. Assuming this sweat composition to be the same as ASW, the deposited mass per deposition time of this study would correspond to palm-surface contact of approximately 70 people.

**Fig 5:**
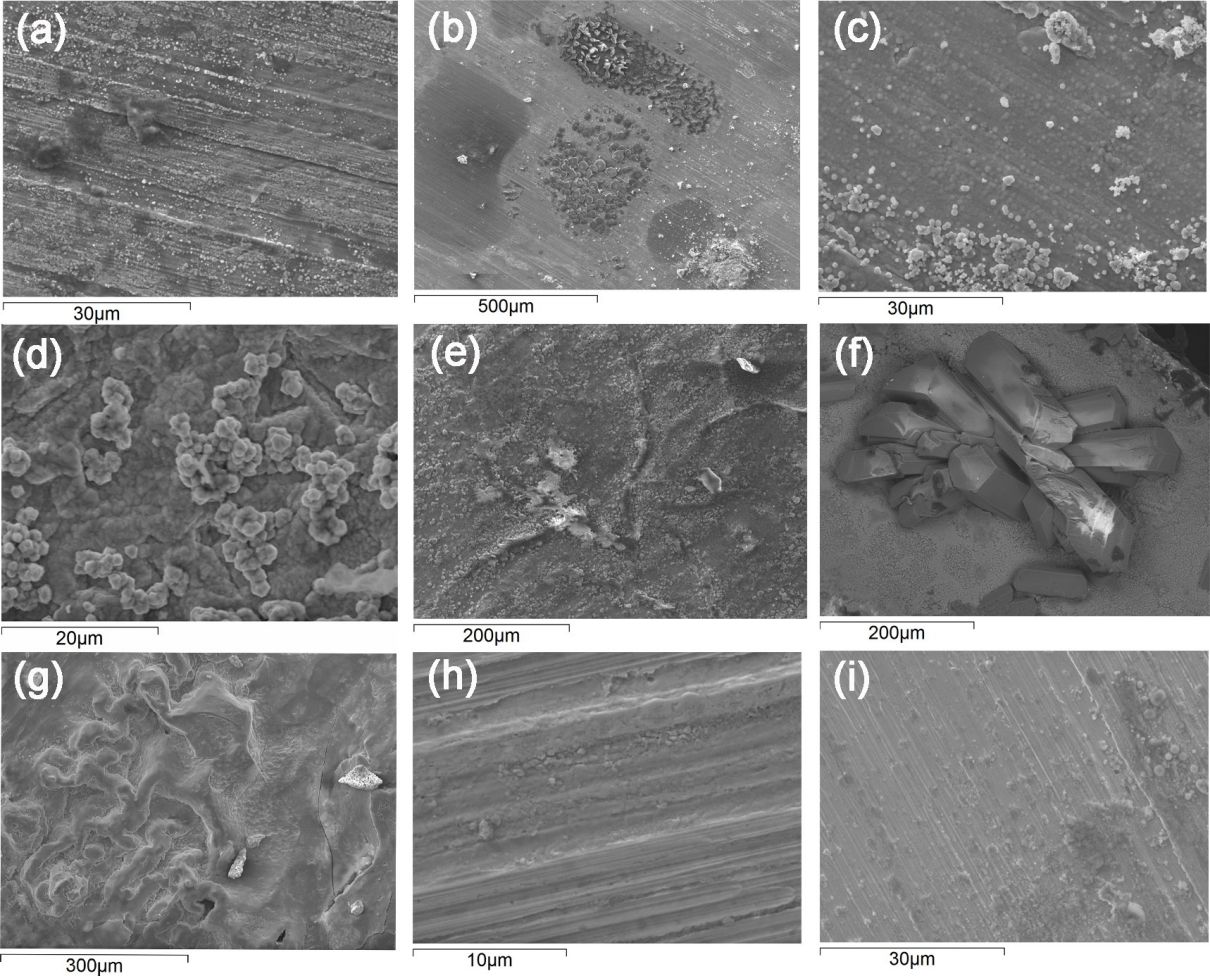
Selected SEM-SE top-surface images of the corrosion product layer of Cu coupons after 2 weeks of daily fingerprint contact and continous daily cycles of repeated dry/wet periods at constant temperature (a) compared with parallel exposures with daily deposition of ASW on Cu metal coupons after 1 day (b-d) and 4 weeks (e-g) and non-ASW-deposited Cu metal coupons after 1 day (h) and 4 weeks (i) of exposure in the climatic chamber.

Artificial sweat was sprayed on a daily basis onto Cu metal coupons followed by their continuous exposure in a climatic chamber at cyclic wet/dry conditions at constant temperature for 1 day, 1, 2 and 4 weeks to simulate (and accelerate) different extent of indoor atmospheric corrosion and finger-print contact. Similar to the occurrence of corrosion features locally distributed on the tarnished/corroded Cu coupons after repeated fingerprint contact, e.g. after 2 weeks of daily thumb imprint and daily dry/wet cycles in the climate chamber for the same time period, Fig 5A, the one-day exposed surfaces, Fig 5B, also displayed corrosion products locally concentrated to the areas of the evaporated aerosol droplets. With time these corrosion products completely covered the surface, Fig 5C–5G. Based on GI-XRD, EDS and FTIR measurements the tarnished/oxidized surfaces were predominantly composed of Cu_2_O and CuO (similar as for the fingerprinted coupons), Fig 5C and 5D with the local presence of chloride-rich corrosion products, possibly Cu_2_Cl(OH)_3_. Surfaces exposed for 4 weeks showed the local presence of Na_2_Cu(CO_3_)_2_×3H_2_O (Chalconatronite), Fig 5F. Compared with the non-ASW-deposited surfaces, spraying and deposition of the ASW aerosol droplets clearly enhanced the corrosion process, here illustrated with top-surface images of non-ASW deposited Cu surfaces exposed for 1 day and 4 weeks in the climatic chamber showing substantially less corrosion, Fig 5H and 5I. All corroded surfaces were assessed on their antimicrobial properties in order to evaluate any effects of the extent of tarnishing/corrosion and finger-print contact, mimicked via deposition of artificial ASW.

Since the one-day Cu metal coupons deposited with ASW showed locally occurring corrosion features, similar to the finger-print conditions, these and the non-ASW-deposited surfaces were used (in parallel to studies on glass slips) to elaborate a general methodology able to mimic the local formation of corrosion products induced by fingerprint contact and hence the local transfer of bacteria to high touch surfaces.

**Fig 6:**
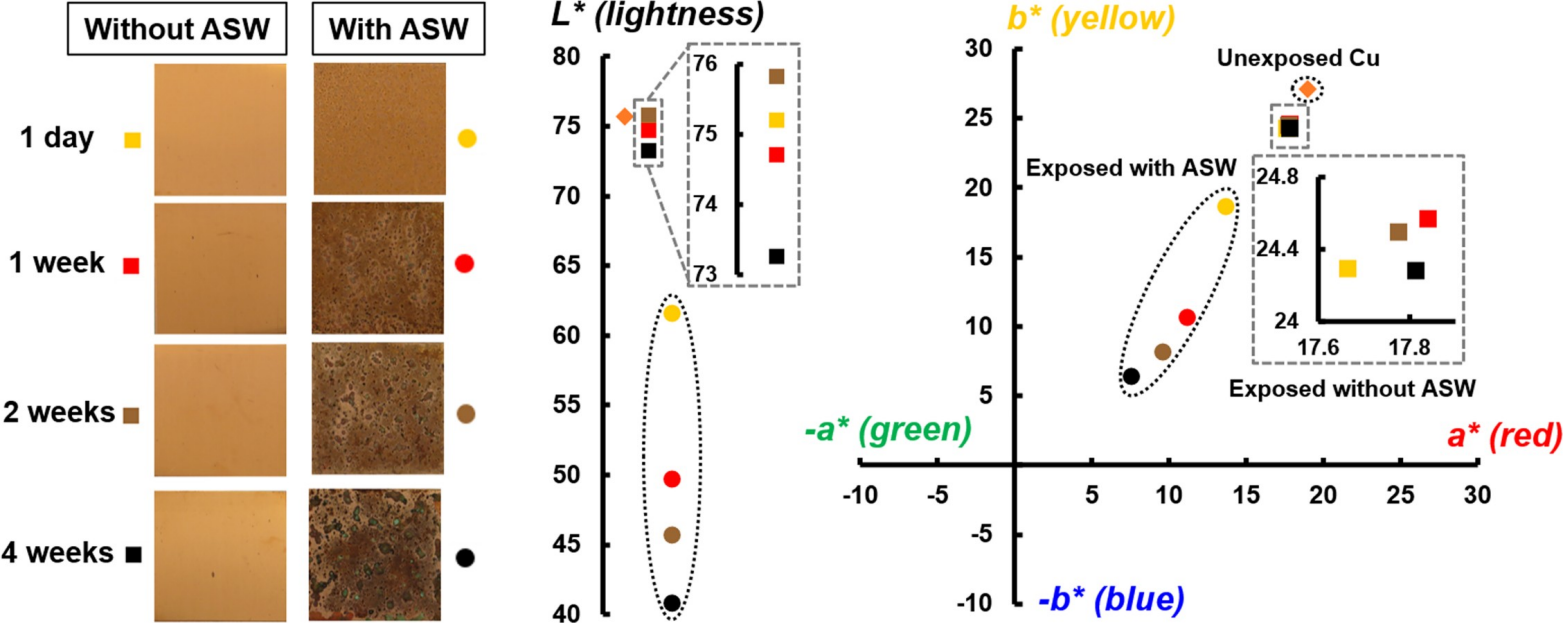
Changes in surface appearance (CIELab color reference space) of Cu metal with and without deposited ASW after climatic chamber exposure at daily repeated dry/wet periods for 1 day, 1, 2 and 4 weeks.

Corrosion/oxidation processes of metallic surfaces typically proceed at a low rate at indoor conditions [38] but can evidently be enhanced upon e.g. finger-print contact, here simulated with ASW aerosol deposition. Such interactions change also the aesthetic surface appearance that may be of large importance for a given application, e.g. for an IH surface. Changes in surface appearance of the differently corroded/oxidized Cu metal coupons as a function of time and deposition of ASW were assessed spectrophotometrically. The results are visualized in Fig 6 by means of light optical micrographs and schematic descriptions of changes in colorimetric parameters. Substantial changes in appearance (ΔE_ASW_: 16–45) were observed between the differently corroded Cu metal surfaces deposited with ASW and non-exposed Cu. Cu coupons deposited with ASW resulted in a substantially reduced lightness (20–33%) compared to parallel exposures without any deposition of ASW and to non-exposed Cu. Repeated dry/wet exposure conditions during 1 day up to 4 weeks in the climatic chamber without any deposition of ASW resulted in minor changes with time (ΔE_NO ASW_: 1.8–4.8) in surface appearance and lightness. These findings are in agreement with previous observations on Cu metal that report loss in lightness related to the thickening of the surface oxide (cuprite) [58]. However, in the presence of ASW, pronounced differences were gradually becoming evident (yellowish-red after 1 day and brownish after 1 week of exposure), Fig 6. Locally occurring blackish and greenish features appeared after longer exposure times (2 and 4 weeks) with reduced lightness and significant offset from the yellow/red quadrant towards the green/blue quadrant, indicative of the local formation of Cu(I)- and Cu(II)- containing corrosion products respectively (58), such as Cu_2_O and Cu_2_(OH)_3_Cl [39, 40].

**Fig 7:**
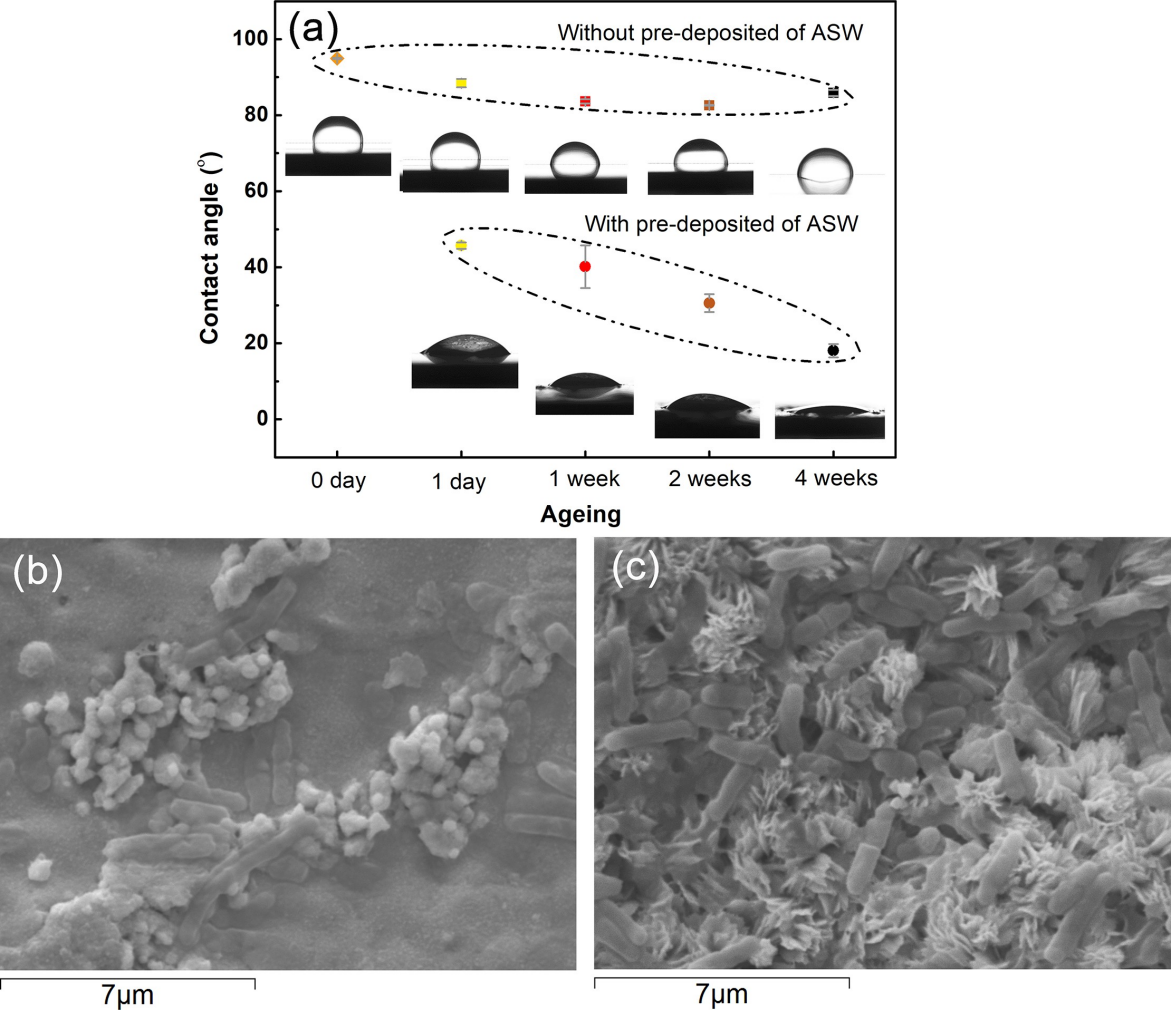
Changes in contact angle (indicative of increased surface wettability) of ASW droplets with time on Cu metal surfaces deposited with and without ASW and exposed to daily repeated dry/wet cycles for 1 day, 1, 2 and 4 weeks (a) SEM images of (b) bacteria and some corrosion products on the surface of Cu metal with deposited ASW for 1 day and (c) bacteria and corrosion products on Cu metal deposited with ASW for 4 weeks.

Corrosion/oxidation of the Cu coupons with and without ASW deposition evidently resulted in corrosion products of different surface coverage, morphology and composition, conditions that influence the surface roughness, which strongly influence the surface wettability that may be used to predict the bacterial adhesion capacity of a given surface (10). Measurements of surface wettability were therefor performed using contact angle measurement to gauge differences in surface-free energy. Changes in contact angles and drop characteristics are presented in Fig 7A for the tarnished/corroded Cu surfaces deposited with and without ASW. Large contact angles reflect a surface of hydrophobic character, while lower values present improved liquid adhesion properties [10], i.e. an improved capacity for bacterial cell adhesion. Slightly reduced contact angles with time were observed for the Cu coupons exposed without any ASW, Fig 7A, whereas the contact angles of coupons deposited with ASW readily dropped with 50% - 80% and continued to be reduced with time. The results follow the same trend with time and extent of corrosion product formation as observed in the surface appearance measurements, and show that the Cu metal surfaces that initially were very hydrophobic, gradually became more hydrophilic and hence more susceptible for bacteria adhesion. This was qualitatively confirmed by the SEM investigations showing an increased extent of bacteria with increasing extent of corrosion, Fig 7B and 7C.

In short, deposition of ASW on the Cu metal surfaces resulted in noteworthy changes in surface appearance, wettability, corrosion product composition and formation rates, as well as surface roughness, parameters that all may influence the antimicrobial properties of the surface. The results highlight the importance to study the antimicrobial properties not only on unexposed bare Cu surfaces but also aged (tarnished/corroded) surfaces in order to assess their applicability for high touch surface applications.

#### Simulation of the transfer of bacteria from fingerprints to a high touch surface

The preferential way to introduce bacteria to the surface in a reproducible way would be via spraying of an aerosol of ASW containing bacteria, as previously employed to deposit ASW droplets for the indoor oxidation study presented above. However, as this would result in uncontrollable contamination of bacteria on different surfaces (within the climatic chamber, the spray nozzel and vessels etc), these aspects had to be controlled and the transfer of bacteria to the surfaces performed in a different way. Different approaches were employed on bare Cu, one-day oxidized Cu with and without ASW, as well as on glass slips using the same strategies as presented above, Fig 2 in Section 2.4.

**Fig 8:**
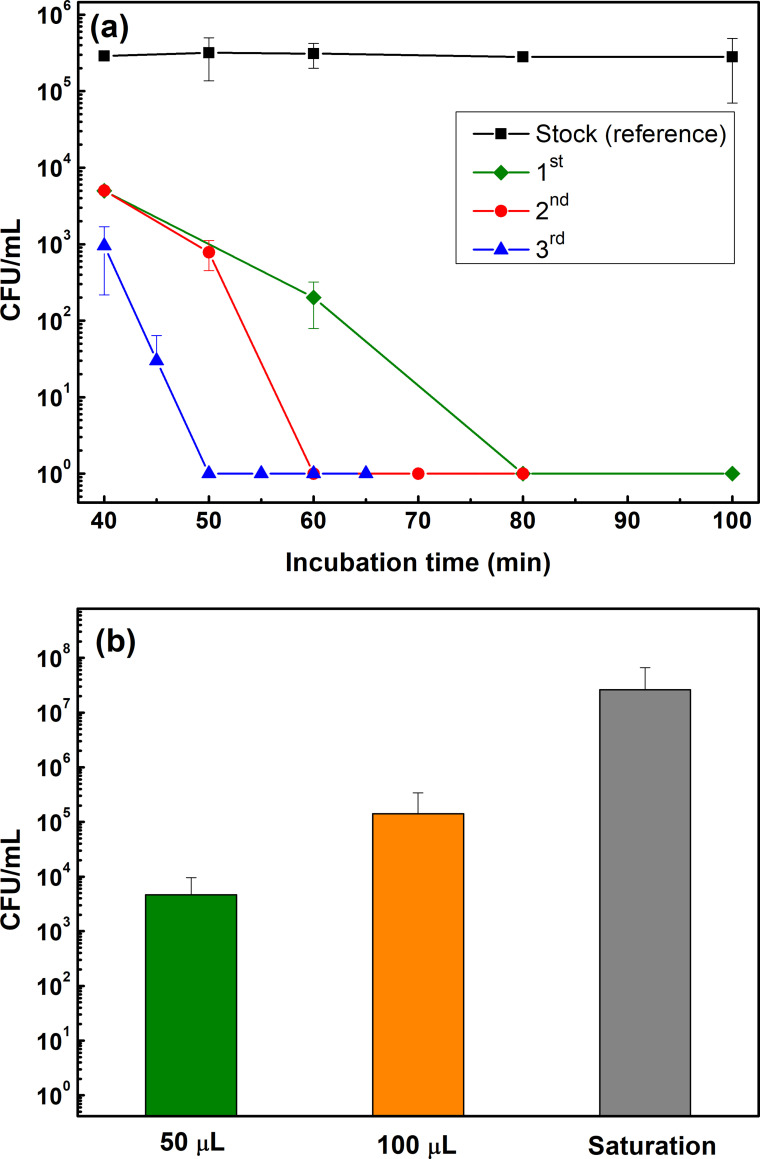
Kinetics of the variation in transferred number of bacteria onto one-day tarnished/corroded Cu surfaces (3 different surfaces, (1^st^, 2^nd^, 3^rd^)) following the wet protocol (a) and the reduction in bacteria (E. Coli) viability in ASW following the dry (b) protocol. Each point reflects the average value based on triplicate coupons for each measurement done three times.

The first approach was based on *wet protocol* by depositing 20 μL ASW containing *E*. *coli* (OD_600_ = 0.5) as small drops with a pipette that were spread over the tarnished/corroded Cu- and control glass surfaces. This procedure was repeated three times (each time on triplicate surfaces) over a period with largely deviating results as illustrated in Fig 8A. Despite an approximately two orders of magnitude reduced number of bacterial colonies after 40 min, the time for complete inhibition of the cell viability varied between 50 and 80 min for the one-day oxidized Cu surfaces at ambient room temperature. This was attributed to the poor reproducibility of the experiment in surface spreading of the bacterial cells. This could mainly be due to uneven distribution of bacterial cells on the surface, low surface wettability (Fig 7) combined with large variations in thickness/surface area coverage of the ASW/bacteria solution, and the long evaporation times (60–80 min).

In the second approach, denoted the *dry protocol*, a sterile cotton swab containing ASW containing bacteria (*E*. *coli*, OD_600_ = 1) either with 50 μL or 100 μL, or until completely soaked (saturated), was used to transfer bacteria to the Cu and glass surfaces. Despite rigorous strategies to swab the surface in a defined way, the number of transferred bacteria was non-reproducible (varying at least one order in magnitude), Fig 8B. This method might deposit a low amount of solution that results in an even distribution of bacteria on the test surface thereby enhancing quick evaporation. The results depicted at least one order of difference in the amount of bacterial cells at the surface, both at each time of deposition and at time t = 0.

**Fig 9:**
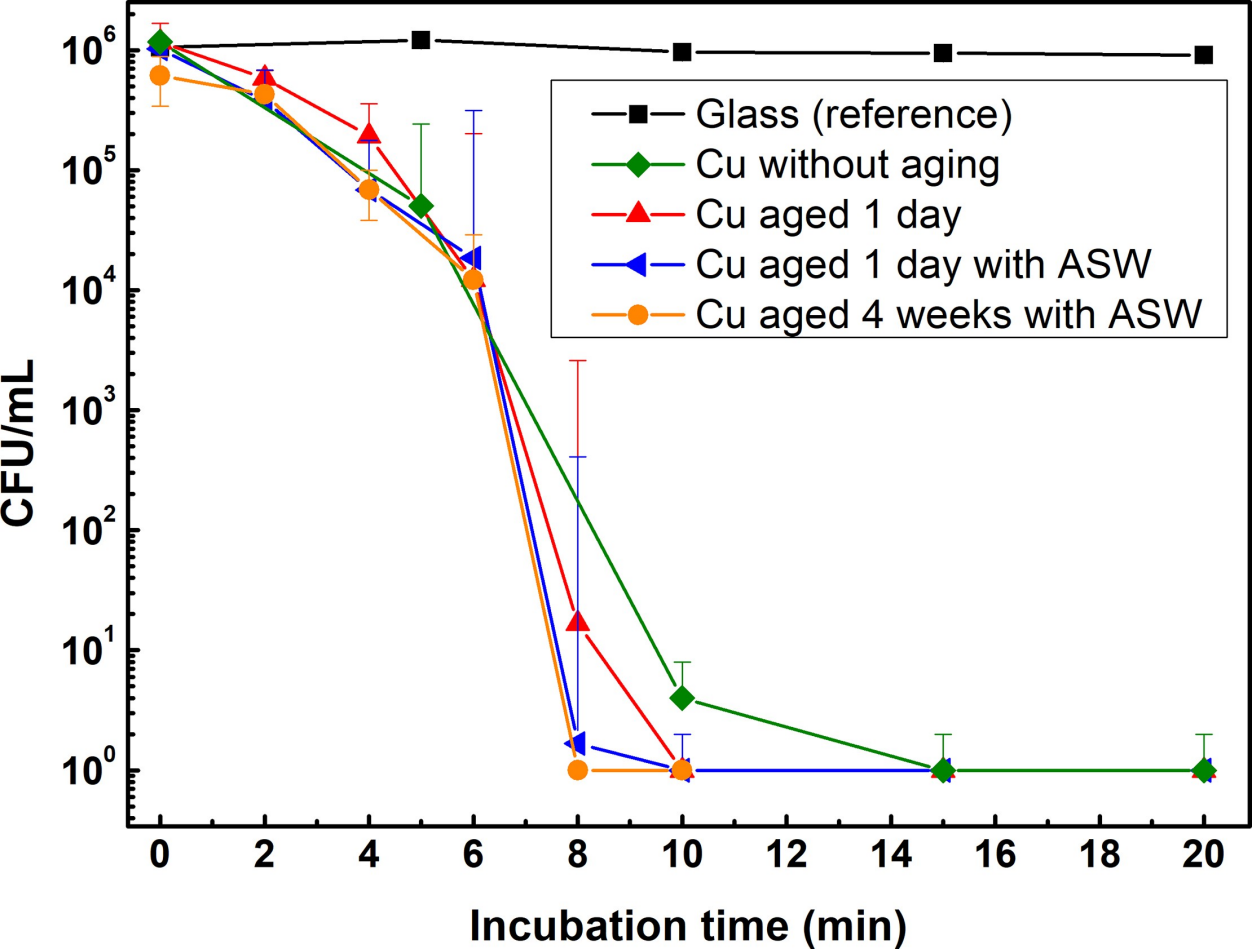
Kinetics in reduction of bacteria (E. coli) viability for one-day and 4 weeks tarnished/corroded Cu metal surfaces following the quasi-dry protocol up to 20 h of incubation at 37°C. Each point is the average value of 6 NA plates including 2 replica from 3 different coupons and the statistic p-values were at all conditions < 0.05 (see detailed values in S2 Table).

The third approach, denoted the *quasi-dry protocol*, was elaborated based on experiences from the two unsuccessful approaches described above aiming to transfer reproducible and similar concentrations of bacterial cells onto the surface for each deposition with sufficiently small ASW sample volumes that readily evaporates within a short time frame (thereby mimic indoor exposure conditions during fingerprint contact). Three micro-liter of ASW containing *E*. *coli* (OD_600_ = 1) were deposited using a micro-pipette onto differently tarnished/corroded Cu metal and glass surfaces (1×1 cm^2^). This approach enabled reproducible droplet volumes to be deposited and the formation of a thin aqueous layer within a few minutes which evaporated substantially faster (within 6±1 min) compared with the wet protocol procedure (65 ± 15 min) described above. The measured numbers of bacteria transferred to the surface (CFU/mL) were in the same order of magnitude at time 0 for both Cu metal and glass (2.34·10^6^ and 2.5·10^6^, respectively) and comparable to the concentration of the stock concentration (3.56·10^6^), Fig 8. Almost a complete reduction in the number of viable bacteria was obtained after 10 (aged Cu) and 15 (as-received Cu) min. The cell viability was reduced with two orders of magnitude in 6 min (during the visible surface drying period) followed by another four orders of magnitude reduction during the following 2 min and almost complete inhibition, (i.e. no viable bacteria left) after 10 min for the corroded and aged Cu surface deposited with ASW (Fig 9). The critical time difference for bacterial cell inhibition among the Cu surfaces aged/corroded for 1 day and 4 weeks with and without ASW occurred in 6 to 10 min of exposure.

Even though the tarnished/corroded Cu surfaces with and without deposited ASW revealed differences in extent and composition of the corrosion products, their antimicrobial capacities were similar, Fig 9. Observed kinetic differences for complete inhibition of the bacteria between the wet and quasi-dry conditions are related to the faster drying time of the thinner ASW layer in the latter case, which both promotes the corrosion process, and thereby the release of Cu ions into the aqueous layer, as well as increases the concentration of bacteria (and corrosive species) in contact with the surface.

In all protocols, the detachment of weakly attached viable and dead bacteria was enabled via vigorous vortexing of the exposed Cu- and glass surfaces in 2 mL ASW for 1 min, followed by instant serial dilution plating on nutrient agar and 24 h incubation to assess any presence of live bacteria detached from the surface. This methodology was selected as it showed the highest possible detachment of *E*. *coli* (OD_600_ = 0.5) from the glass surfaces exposed for 20 h in nutrient broth compared to the other detachment techniques investigated including i) vigorous vortexing (1 min), ii) vortexing with glass beads (1 min), iii) sonication in ultrasonic bath (30 s), and iv) mixing using a rotator with 3 mM SDS (sodium dodecyl sulfate) for 30 min. No viable bacteria were observed on Cu metal exposed in parallel. The detachment procedure was immediately followed by impressing the Cu- and the glass surfaces onto NA plates in order to assess any viable bacteria still adherent on the surfaces (S1 Table). Following the imprint, no viable bacteria were present on any of the Cu surfaces, independent on detachment treatment.

**Fig 10:**
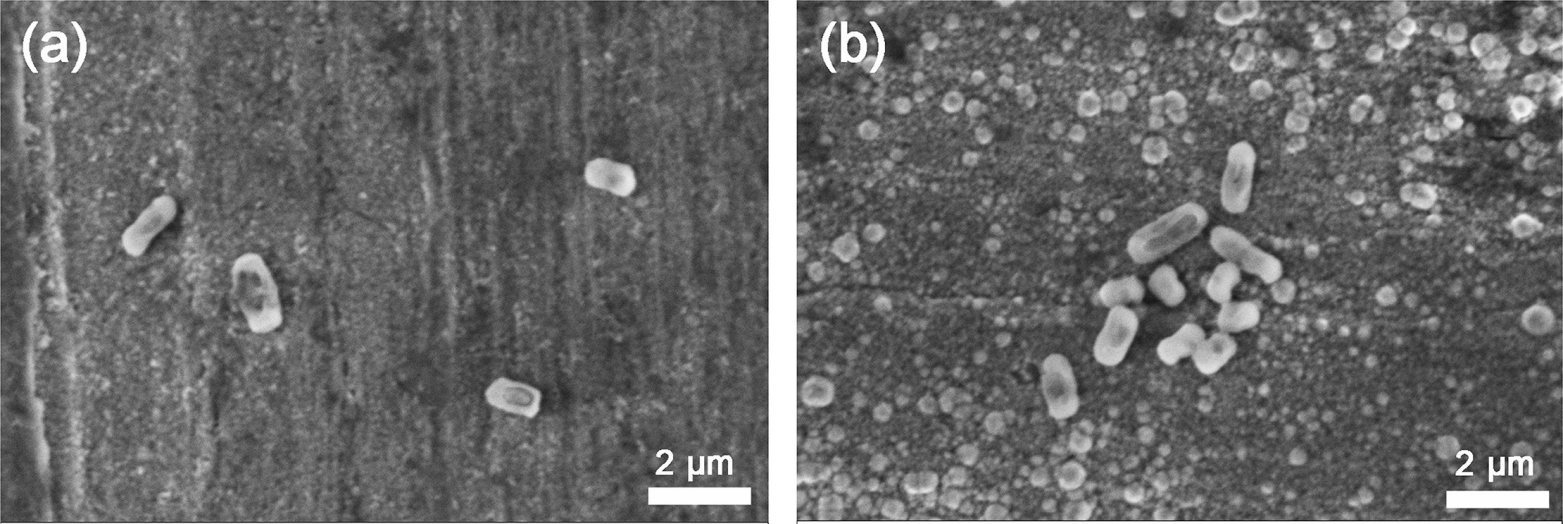
SEM images of surface morphologies and presence of bacteria adherent to the surface of one-day tarnished/corroded Cu metal without (a) and with (b) ASW deposition followed by exposure for 24 h to E. Coli (OD_600_ = 0.5) and the concomitant detachment of looosly adherent bacteria via vigorous vortexing (1 min) in ASW.

The efficiency of the bacterial detachment from the Cu surfaces by the vortexing method was examined by means of SEM. As illustrated in Fig 10, some bacteria remained on the surfaces, suggesting their strong surface adhesion. A higher number of bacteria remained on the one-day oxidized Cu surface deposited with ASW compared with non-ASW-deposited surfaces, an effect most probably related to higher surface roughness and hence higher wetting abilities (see Fig 7). The presence of adherent bacteria (viable and/or dead) may act as local corrosion cells that result in crevice conditions that in turn may lead to localized corrosion. Studies of these aspects are on-going. Due to the high vacuum and electron beam of SEM, it was not possible to judge with this technique whether the strongly adherent bacteria were dead or viable.

#### Staining to assess the presence of dead and viable bacteria with time on high touch surfaces

The viability of adherent bacteria, monitored as changes in membrane integrity, on freshly prepared one-day pre-oxidized Cu surfaces with time was analyzed using a viable/dead staining assay combined with confocal fluorescence microscopy imaging. Dead and viable bacteria are stained red and green, respectively.

**Fig 11:**
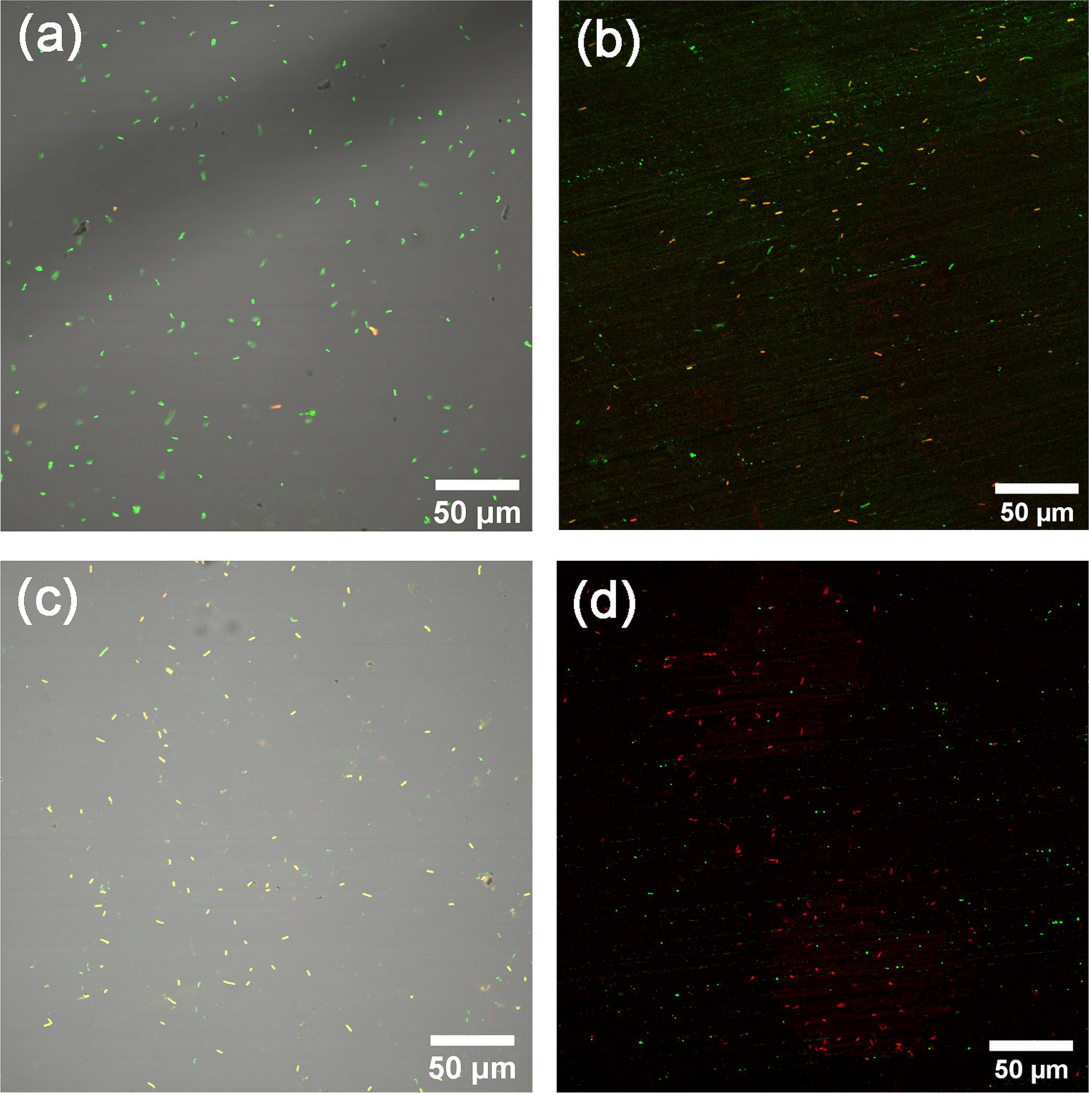
Representative fluorescence microscopy images of viable/dead bacteria on glass with merged transmitted light (a, c) and on one-day tarnished/corroded Cu surfaces (b, d) exposed to E. coli (OD_600_ = 0.9 ± 0.1) and stained with an viable/dead staining assay after 0 min (+10–15 min imaging time) (a-b) and after 20 min (+10–15 imaging time) (c-d). Live (green) and dead (red) cells were dyed by means of SYTO9 (a green fluorescent nucleic acid stain) and PI (propidium iodide, a red-fluorescent nuclear and chromosome counterstain).

In order to assess the reduction in the viability of *E*. *coli* upon contact with the differently tarnished/corroded Cu metal surfaces in real time, live and dead staining method was followed. Fig 11 shows viable bacteria without any cell membrane damages on the glass surface, while some bacteria were damaged on the Cu surface already at time 0 min (i.e. in reality 10–15 min due to the imaging time), indicative of a rapid antimicrobial capacity. After 20 min, the majority of the bacteria on the Cu surfaces displayed damaged cell membranes. Some damaged cells were also observed on the glass surface after the same time period, though most probably related to general desiccation conditions with time [61]. It should be mentioned that the Cu surface may interact with SYTO9 causing green fluorescence residues, i.e. some green dots on Fig 11B and 11D, see S1 Fig for a clearer identification). These real-time measurements are in agreement with the antimicrobial property findings observed for the tarnished/corroded Cu surfaces when following the quasi-dry protocol described above, Fig 9. This supports the applicability of the elaborated protocol to study antimicrobial efficiencies and effects of tarnishing/corrosion of high-touch surfaces of relevance for IH applications at laboratory conditions.

In short, the elaborated modus of operandi of the quasi-dry protocol to transfer bacteria and study the antimicrobial properties of different high-touch surfaces was shown to be more reproducible and to more realistically mimic fingerprint contact (here simulated with ASW droplets) compared to existing wet and immersion condition test protocols available in the scientific literature.

### Copper release

The antimicrobial function of Cu metal and its alloys has in the literature been connected with the release/dissolution of Cu ions immersed in solution [7, 9, 17, 56]. However, since such conditions are very different from the aqueous thin film present at atmospheric conditions both at outdoor and indoor conditions [38, 62], their relevance for high-touch surfaces and IH applications can be argued.

**Fig 12:**
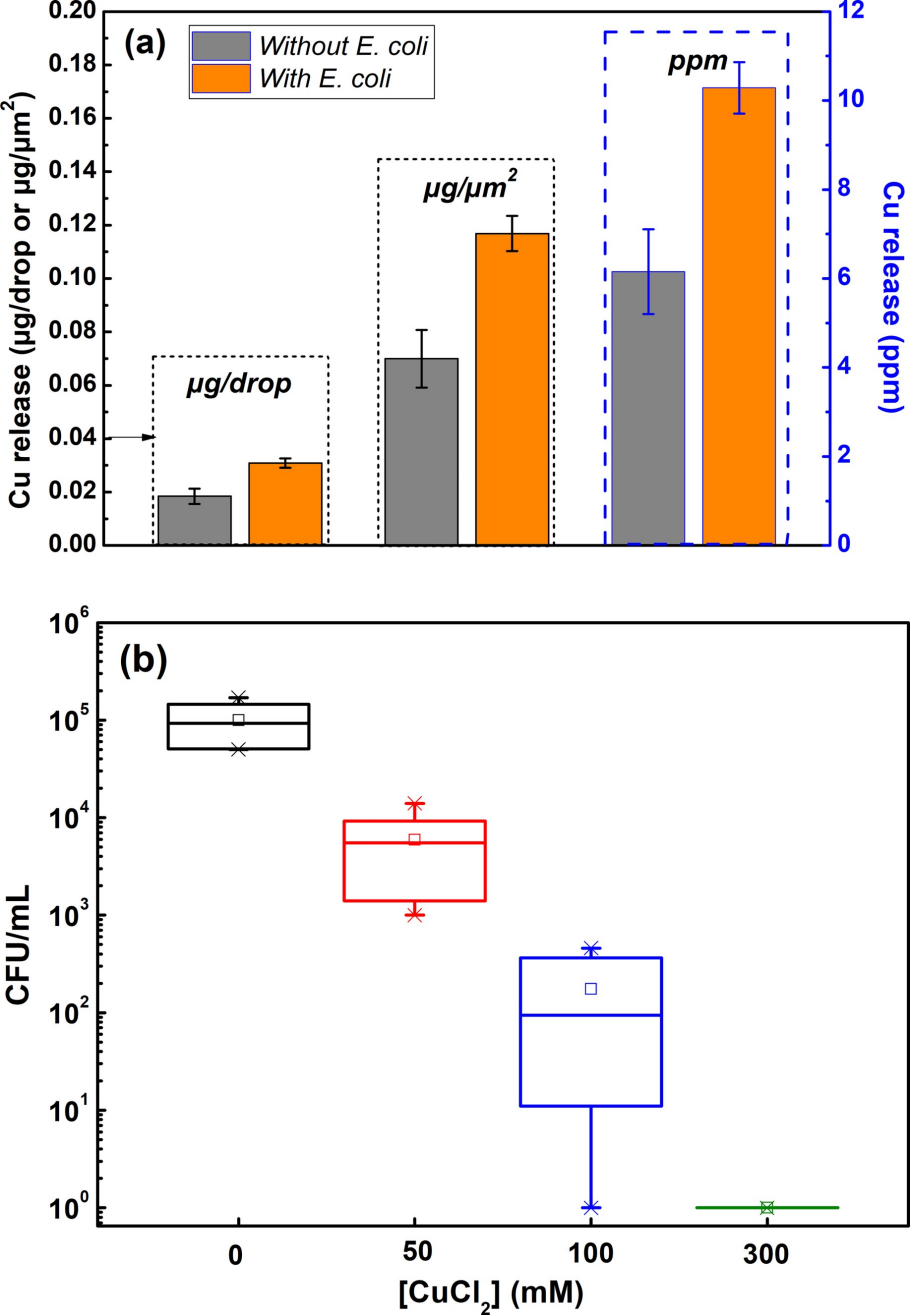
Released amounts of Cu in deposited ASW droplets (following the quasi-dry protocol) after 10 min of exposure of one-day aged (tarnished/corroded) surfaces both with and without the presence of E. coli (a); bacterial survival (p = 0.04 < 0.05) of E. Coli (OD_600_ = 1) after 10 min of exposure in ASW solution at different Cu^2+^ concentrations (b).

Since complete inhibition of the *E*. *coli* viability was obtained after 8–10 min when tested according to the quasi-dry protocol, see Fig 9, droplet release studies were performed for 10 min on one-day aged (tarnished/corroded) surfaces both with and without the presence of *E-coli*. The released amount of Cu per droplet (≈1:1 area to solution volume ratio) was determined by means of AAS and assessed as the mean value of in total 192 droplets per coupon (in total 576 droplets on triplicate surfaces). The results, presented in Fig 12A, show high amounts of released Cu /surface area (6 orders of magnitude more compared with the background) into the droplets (0.07 ± 0.01 μg/cm^2^) and the presence of bacteria (OD_600_ = 0.1) to induce almost twice as (1.7 times) high released amounts of Cu per droplet (0.12 ± 0.01 μg/cm^2^). Higher released amounts of Cu in the presence of bacteria are speculated to be a consequence of the formation of crevice conditions between adherent bacteria and the tarnished/corroded surface that disables the protective properties of the surface oxides, an aspect to be investigated in future studies.

Parallel studies with known concentrations of CuCl_2_, performed to assess the concentration necessary to kill the bacteria (although conducted at 10 times higher bacteria concentration), showed a reduction of the bacteria viability by one order of magnitude when performing the quasi-dry protocol adding 50 mM (≈3000 ppm) Cu (as CuCl_2_) to the bacteria solution. This is 4 orders of magnitude higher concentrations than measured in the vortexed solution (area to solution volume ratio ≈2:1) of the one-day aged and exposed coupons (0.55 ppm) to *E-coli* for 10 min following the quasi-dry protocol (after coupon and bacteria removal). The bacteria viability was reduced by 2 orders of magnitude when adding 100 mM Cu, and complete reduction was obtained when adding 300 mM Cu (see Fig 12B). These results imply that the antimicrobial properties depend not only on surface characteristics (as described above) but also to the extent of released Cu ions. The difference in cell viability in quasi-dry and liquid environments could be due to the contact of Cu ions with bacterial cells. When the cells are immersed in CuCl_2_ solution, the bacteria are dispersed and in planktonic forms which might not have a direct contact with Cu ions whereas when the bacterial cells, when exposed to Cu surface in the quasi-dry method, are in close contact with the surface and thereby have direct access to chemical interactions which enhance membrane damage and further cellular consequences. This might influence the concentration required for killing bacterial cells.

## Concluding summary

A novel methodology for combined studies of tarnishing/corrosion and antimicrobial properties of high-touch surfaces mimicking indoor atmospheric conditions (governed by thin aqueous layers) and fingerprint contact has been elaborated using Cu metal as benchmark material. Fingerprint contact was simulated by adding bacteria onto differently tarnished/corroded surfaces with and without deposited artificial sweat (ASW) droplets. The influence of surface characteristics and reactivity, including tarnishing/corrosion, surface roughness, surface wettability, Cu release and formation of corrosion products was assessed from an antimicrobial perspective.

**Fig 13:**
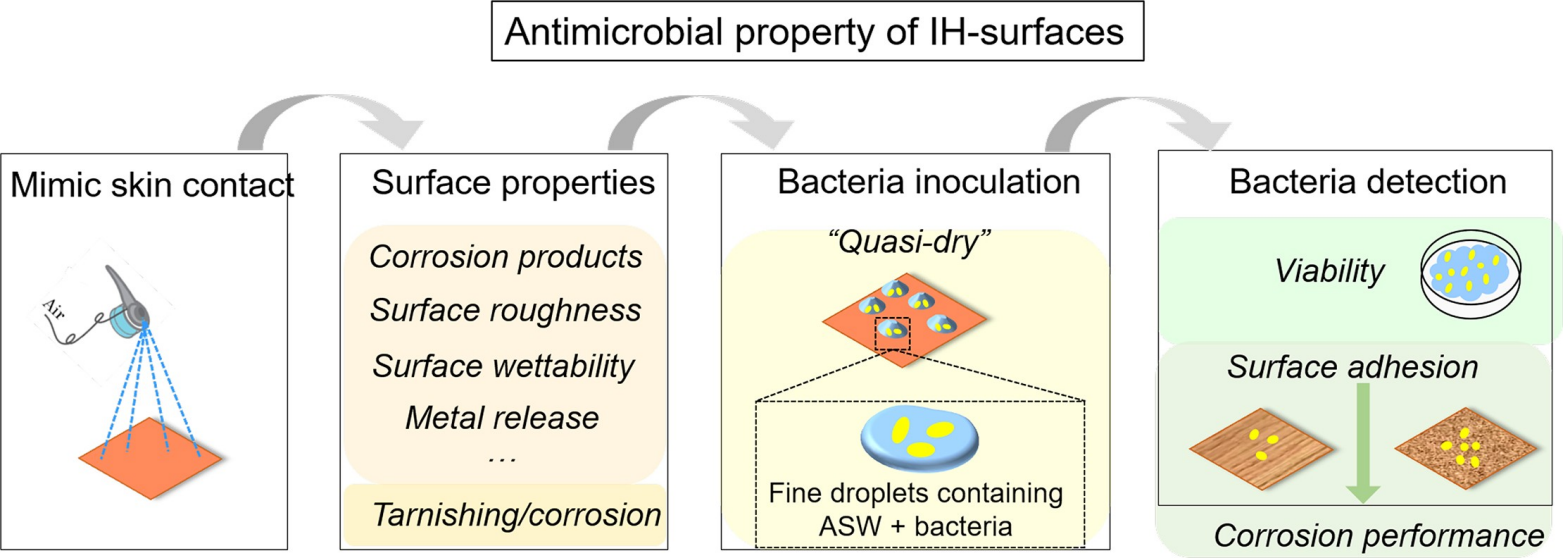
Schematic illustration of the test methodology and investigated properties to assess the surface characteristics and antimicrobial properties of high-touch surfaces. Cu metal was used as benchmark material in this study.

The approach and investigated properties are summarized in Fig 13.

The methodology, denoted the quasi-dry protocol, was elaborated in which bacteria was introduced to the high touch surface via well controlled initially locally separated ASW droplets that formed a thin aqueous layer within a few minutes and that evaporated substantially faster (< 6 min) compared with conditions obtained using e.g. existing wet protocols (50–80 min) which rather mimic immersion conditions. The quasi-dry protocol resulted in a substantially improved reproducibility both in respect to bacteria transfer as well as viability effects compared to reported [33, 63] and tested wet and dry protocols (Figs 8 and 9). Generated results following the quasi-dry protocol revealed rapidly reduced viability of *E*. *coli* (within 6 to 10 min), an effect most probably enhanced by the rapid release of Cu ions (Fig 12A) within the ASW droplets, and from increased concentrations of Cu ions during the evaporation of the droplets and the aqueous layer. High concentrations of Cu ions in contact with the bacteria can, as suggested in the literature [19, 32, 61], via contact killing induce cell damage, Cu influx into the cell, DNA damage and cell death, and thereby enhance the antimicrobial effects of the Cu metal surface.

Except for inducing changes in aesthetic appearance (Fig 6), indoor tarnishing/corrosion as well as repeated mimicking of finger-print contact (ASW droplet deposition) resulted in an increased extent of corrosion products of different composition (predominantly Cu_2_O, CuO and to some extent for the ASW-exposed surfaces also Cu_2_Cl(OH)_3_ and Na_2_Cu(CO_3_)_2_×3H_2_O). From this followed an increased hydrophilicity (Fig 7) and increased surface roughness (Figs 6, 7 and 10), parameters that influence the probability of bacteria adhesion, in particular for the most corroded surfaces (Figs 7 and 10). However, no evident effects by corrosion product composition were observed on the antimicrobial efficiency (Fig 9). Literature findings report slightly different antimicrobial efficiencies of tarnished/corroded Cu metal with a surface composition of either Cu_2_O or CuO (35). It is also suggested that the solubility of the corrosion products can provide a qualitative measure of its antimicrobial function [64], i.e. the more soluble corrosion products, the higher antimicrobial efficiency. However, as the corrosion products often contains several compounds at ambient atmospheric conditions, as observed in this study, it can be difficult to relate observed differences in antimicrobial properties to a specific compound. Results of this study, generated at immersion conditions (S2 Fig), showed increased concentrations of released Cu (after 4h) with an increased extent of corrosion products (and of different composition) for surfaces daily exposed to ASW, whereas similar extent of released Cu was observed for the differently tarnished/corroded surfaces exposed at repeated dry/wet cycles only (S2 Fig). However, the lack of observed relation between the antimicrobial efficiency and the extent of corrosion/corrosion products or their composition (Fig 9) is most likely attributed to sufficiently high released concentrations of Cu within the ASW droplets (both with and without bacteria present), which in all cases resulted in complete reduction in bacteria viability already within 8–10 min (Figs 9 and 12).

This study shows that differences in corrosion product formation and composition alter the physical properties (e.g. surface wettability, surface roughness) of a high-touch surface (Figs 6 and 7). Since the probability of bacteria adhesion is promoted by increased surface roughness (Figs 6, 7 and 10), the possibility for direct contact killing increases. However, since a higher surface roughness also implies a higher risk for bacteria contamination of the surface, hydrophobic materials (contact angles > 65°) are usually recommended for antimicrobial applications since such surfaces hinder bacteria attachment [10]. It should nevertheless be stressed that different bacteria and strains have different hydrophobic/hydrophilic- and adhesion properties [65].

The presence of adherent bacteria and their secreted extracellular polymeric substances are expected to influence the corrosion performance (e.g. induce localized corrosion) of a Cu metal surface (Fig 10) and consequently also influence its antimicrobial properties [5, 66]. These aspects are considered in an on-going study by the authors as well as the importance of efficient cleaning and disinfection for the antimicrobial efficiency of high-touch surfaces [2, 43, 67, 68].

In all, the antimicrobial efficiency of a high-touch surface depends on both physical and chemical properties of a material. Although this study has not investigated the mechanisms behind observed antimicrobial effects of Cu metal, it proposes a novel general reproducible methodology at laboratory conditions on how to study the antimicrobial properties of high-touch surfaces mimicking fingerprint contact and indoor atmospheric corrosion.

## Supporting information

S1 Fig Representative fluorescence microscopy images of viable/dead bacteria on one-day tarnished/corroded Cu surface exposed to E. coli (OD_600_ = 0.9 ± 0.1) and stained with an viable/dead staining assay after 0 min (+10–15 min imaging time). Live (green) and dead (red) cells were dyed by means of SYTO9 (a green fluorescent nucleic acid stain) and PI (propidium iodide, a red-fluorescent nuclear and chromosome counterstain).
(JPG)

S2 Fig Copper ion release from aged Cu surfaces exposed up to 4 weeks with and without pre-desposited ASW after inmmersion in ASW for 4, 24 and 168 h. (TIF)

S1 Table (XLSX)

S2 Table (PNG)
