# Supplementary figures and images for: A novel methodology to study antimicrobial properties of high-touch surfaces used for indoor hygiene applications—A study on Cu metal
Source: PLoS One. 2021 Feb 25;16(2):e0247081. doi: 10.1371/journal.pone.0247081 (PMC7906481; doi:10.1371/journal.pone.0247081)

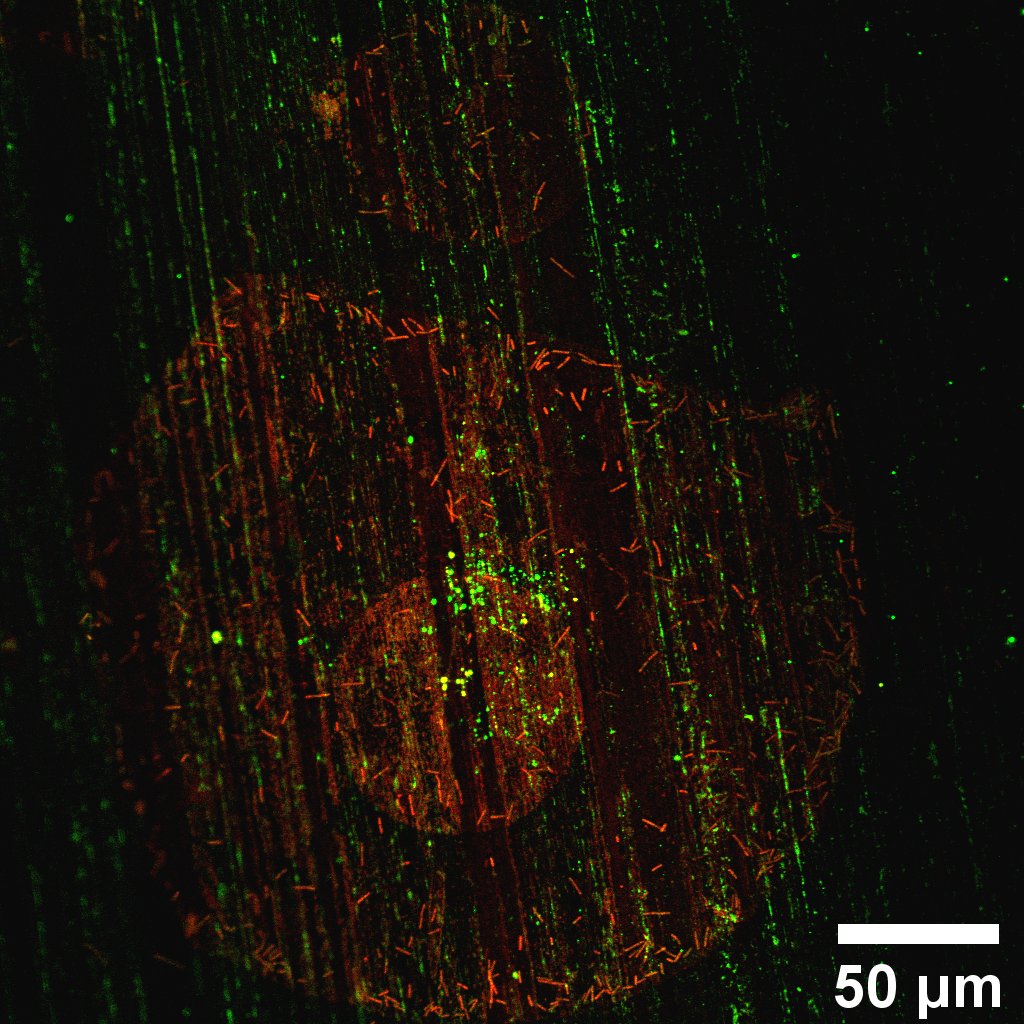

Supplement: S1 Fig — Live (green) and dead (red) cells were dyed by means of SYTO9 (a green fluorescent nucleic acid stain) and PI (propidium iodide, a red-fluorescent nuclear and chromosome counterstain). (JPG) [file pone.0247081.s001.jpg]

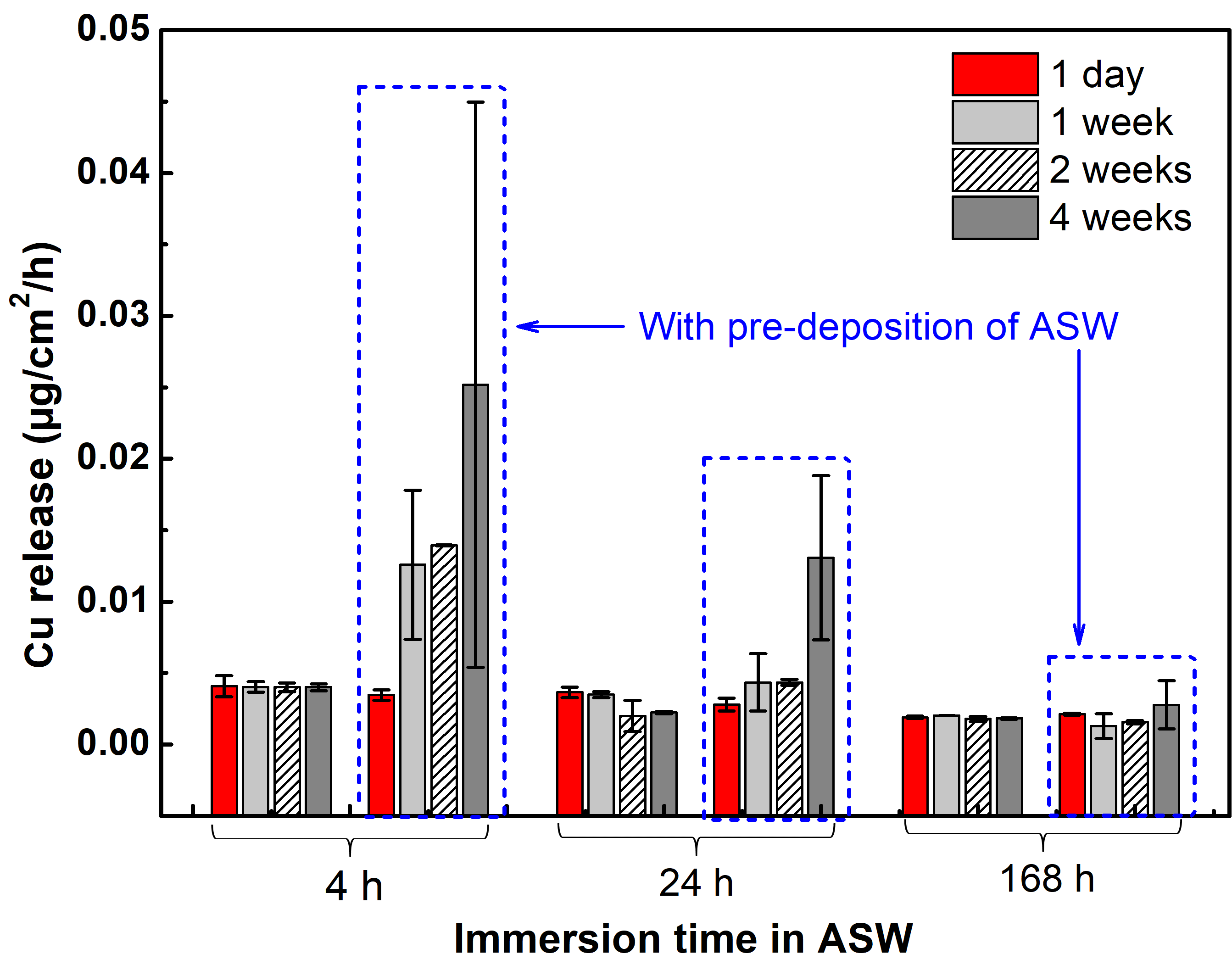

Supplement: S2 Fig — (TIF) [file pone.0247081.s002.tif]

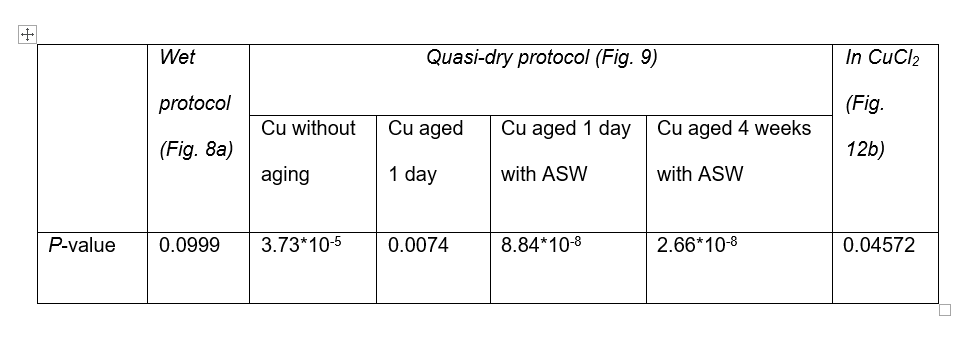

Supplement: S2 Table — (PNG) [file pone.0247081.s004.png]
